# General Molecular Dynamics Approach to Understand the Mechanical Anisotropy of Monocrystalline Silicon under the Nanoscale Effects of Point Defect

**DOI:** 10.3390/nano11081965

**Published:** 2021-07-30

**Authors:** Wei Wan, Changxin Tang, Jianjie Zhang, Lang Zhou

**Affiliations:** Institute of Photovoltaics, Nanchang University, Nanchang 330031, China; 5701118206@email.ncu.edu.cn (W.W.); zjj18894881057@163.com (J.Z.); lzhou@ncu.edu.cn (L.Z.)

**Keywords:** molecular dynamics, monocrystalline silicon, mechanical anisotropy, point defect

## Abstract

Mechanical anisotropy and point defects would greatly affect the product quality while producing silicon wafers via diamond-wire cutting. For three major orientations concerned in wafer production, their mechanical performances under the nanoscale effects of a point defect were systematically investigated through molecular dynamics methods. The results indicated anisotropic mechanical performance with fracture phenomena in the uniaxial deformation process of monocrystalline silicon. Exponential reduction caused by the point defect has been demonstrated for some properties like yield strength and elastic strain energy release. Dislocation analysis suggested that the slip of dislocations appeared and created hexagonal diamond structures with stacking faults in the [100] orientation. Meanwhile, no dislocation was observed in [110] and [111] orientations. Visualization of atomic stress proved that the extreme stress regions of the simulation models exhibited different geometric and numerical characteristics due to the mechanical anisotropy. Moreover, the regional evolution of stress concentration and crystal fracture were interrelated and mutually promoted. This article contributes to the research towards the mechanical and fracture anisotropy of monocrystalline silicon.

## 1. Introduction

Monocrystalline silicon is widely used in the photovoltaic industry and semiconductor production due to its specific photovoltaic effects and semiconductor characteristics. In solar cell production, monocrystalline silicon is fabricated via the floating zone or Czochralski methods [1] first; then, it is sent to the cutting process to produce silicon wafers. Diamond-wire cutting techniques are used to process silicon wafers due to the advantages of low surface damage and high efficiency [2,3]. However, diamond-wire cutting causes edge collapse, hidden crack and surface damage in the wafer fabrication and then results in mechanical problems [4] due to the brittleness and crystal defects of silicon. Meanwhile, it is hard to study the mechanical performance of monocrystalline silicon via experimental methods. Thus, there is a need to understand the mechanical performance of monocrystalline silicon and find out the reasons that limit the quality of wafer cutting. Except for the improvement of diamond-wire cutting itself, mechanical anisotropy [5] is considered as one of the limitations for the optimization of the cutting parameter [6], because the properties related to the orientation could affect the mechanical performance while producing the silicon wafers. For example, the growth of monocrystalline silicon is usually along with the <111> orientation family and the wire saw cutting is perpendicular to the <110> orientation family to minimize product loss. On the other hand, a certain mechanical performance is required to decrease the thickness of the silicon wafers in the application of thin-film solar cells.

From this aspect, many studies have been carried out to reveal the mechanical anisotropy of silicon. Ebrahimi et al. [7,8] studied the fracture anisotropy and crack path in monocrystalline silicon. Brookes et al. [9] investigated the anisotropy of hardness in single silicon crystal. Their results have suggested that the fracture path and toughness were significantly affected by the inclination angle of cleavage planes relative to the indent planes, which also provided a basis for our research. The cleavage fracture anisotropy of silicon was studied by Perez et al. [10], who suggested that cleavage and crack propagation anisotropy of monocrystalline silicon could be explained by lattice trapping. Moulins et al. [11] modelled cracks together with the internal stress analysis of silicon crystal, which gave a deep comprehension of silicon fractography, since the structural orientation was supposed to be the reason for crystal anisotropy. Mylvaganam et al. [12] revealed the deformation behaviors of three typical silicon crystal orientations of [001], [110] and [111]. George et al. [13] investigated several crystal orientations with different cleavage planes and crack fronts and concluded that the anisotropic dislocation movements came from dislocation nucleation and growth. The structural response of silicon was reported via femtosecond laser irradiation [14]; a pronounced amorphization effect was observed in the {111} plane family whereas no disordered structure was detected at the planes close to the {100} plane family. To further test and observe the mechanical performance at the nanoscale, nano-indentation methods combined with numerical simulations were used in the nano-deformation experiments of monocrystalline silicon [15]. Rickhey et al. [16] proposed a model to simulate anisotropic cracking in the Vickers indentation of monocrystalline silicon. The results indicated variations of the crack size for the (001), (110) and (111) planes of monocrystalline silicon. The nano-indentation method has become a powerful research tool for revealing the mechanical properties of monocrystalline silicon at the nanoscale.

Another numerical method is molecular dynamics simulation, which has been used in research about micro-mechanics such as the size and minimum chip thickness effects, elastic-plastic deformation and microstructure effects [17]. Molecular dynamics simulation could provide exciting insights into multiple mechanical problems which are difficult to reveal through experimental methods. Komanduri et al. [18] described the principles of molecular dynamics simulation, relative advantages, current limitations and their application to a range of machining problems. Molecular dynamics simulation could also help us to understand the effects of cutting parameters and the influence of material properties on mechanical processing [19]. Gumbsch et al. [20] investigated dynamic crack stability through molecular dynamics with the results of the systematic form of the crack instability depending on crystal structure, crystal orientation and dislocation generation/motion. Wang et al. [21] discussed the effects of crystal orientation on polishing the non-continuous silicon surfaces, and the conclusions showed that the (010) plane accumulated chips easier than the (011) and (111) planes, and the main phase transformation atoms amount of the (111) plane was the largest among the three planes. The results about crystal anisotropy demonstrated that the mechanical deformation process was affected by the orientation.

There are multiple impurity and defect concerns in the wafer production. The effects on mechanical properties caused by the crystal defects (point defect, dislocation and grain boundary) in the wafer production should be clarified and combined with orientation effects while studying the anisotropy of silicon in depth. In the growth process of monocrystalline silicon, the formation of point defects led to the potential defectiveness [22] in actual application. To study the effects of a point defect, Korsos et al. [23] studied the characterization of a point defect in silicon production and suggested that the defect would cause strength decay in mechanical processing. Menold et al. [24] induced a point defect with an effects test in monocrystalline silicon via spot laser melting, which provided a new method in further study. All of this research has provided the most recent progress for the study of silicon defects in this paper.

There are also some relevant studies for the defect and mechanical anisotropy in other materials like borophene, gold, graphene and black phosphorus [25,26,27]. However, for the mechanical anisotropy of monocrystalline silicon in the molecular dynamics view, the nanoscale effects of a point defect have not been reported yet. Starting with mechanical anisotropy, the effects of a point defect are easier to investigate compared with dislocation and grain boundary because of its zero-dimensional characteristics. Discussions about the inclination angle between the orientation and crystal defect are not required while testing the mechanical performance, which brought certain advantages for the analysis. Thus, a series of molecular dynamics simulations were commenced to study the mechanical anisotropy of monocrystalline silicon under the nanoscale effects of a point defect.

The present paper is a continuation and extension of our previous work [28]. In this paper, the mechanical properties of the [100], [110] and [111] orientations of silicon crystal were systematically investigated via molecular dynamics. Parameters about mechanical performance were calculated for the reference of cutting techniques. The anisotropic fracture phenomena of monocrystalline silicon were presented to evaluate its mechanism of fracture. By generating point defects with different atomic sizes, the nanoscale effects of a point defect on the mechanical anisotropy of monocrystalline silicon were also pointed out. The study of dislocation about the fracture process was presented via dislocation analysis. Visualizations and numerical analysis of the internal stress were provided to indicate the extreme stress region with its geometric and numerical distribution directly. Selected views of different atoms were plotted to discuss the distributions and variations of internal stress in the fracture process. The present paper aims to reveal the mechanical anisotropy of monocrystalline silicon under the nanoscale effects of point defects and provides a reference for the optimization of cutting parameters and the anisotropic fracture mechanism of monocrystalline silicon.

## 2. Model and Method

### 2.1. Model

**Figure 1:**
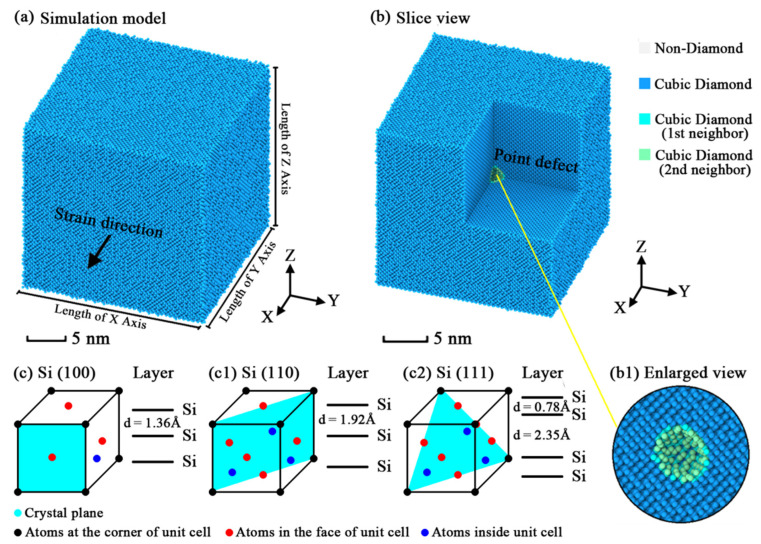
Snapshots of the simulation models: (**a**) snapshot of general simulation models; (**b**) slice view of the point defect; (**b1**) enlarged view of the point defect with a slice along the YZ plane; (**c**) arrangement of atoms at the (100) plane of silicon; (**c1**) arrangement of atoms at the (110) plane of silicon; (**c2**) arrangement of atoms at the (111) plane of silicon.

The simulation models of monocrystalline silicon were generated using the LAMMPS (large-scale atom/molecule massive parallel simulator) software. The lattice constant of silicon for the diamond structure at 300 K is 0.543 nm. Three kinds of simulation model were designed to match the cell structures of designated orientations. The visualization of a typical model is plotted as Figure 1. The atomic arrangements of the three crystal orientations/planes are also indicated. For each model, the X, Y and Z axes and their corresponding crystal orientations with other geometric details are listed in Table 1. The axial length of each model was set as close as possible to control the total count of atoms close to a special value (approximately 500,000 atoms), which would not cause any additional effects on the simulation and could balance the scale of the system with the efficiency of the calculation. The amount of atoms in each model cannot be the same but the most optimal solution, which would not cause non-integer cut-off of crystal cells or additional grain boundaries. By dividing spherical regions with different radius in the geometric center of the models and deleting the silicon atoms inside the regions, the point defects with different sizes were generated to study its nanoscale effects on the mechanical performance of silicon. The size of the point defect in each model is measured by the amount of atoms contained in the spherical region. This measurement is clearer and more direct compared with the measurement of the spherical radius. Six simulation cases with different defect sizes were performed for each kind of simulation model. As there were 18 simulation models, we developed a specific identification system to identify the orientation and defect size of each model. First, the three kinds of simulation model with different orientations were distinguished using the IDs “[100]”, “[110]” and “[111]”, which respectively represent the orientations of the models. With a postfix representing the simulation case behind each ID, the details of the simulation models are clear to readers while reading the present paper. Table 2 shows the representative meanings of the postfixes. For example, ID “[111]-3” means that the X axis of this simulation model is in [111] orientation and it contains a defect with the size of 123 silicon atoms.

### 2.2. Potential

There are over 30 versions of interatomic potential [29] describing the atomic interaction of silicon. However, for the accuracy of simulation, only the potential which best describes the mechanical properties of silicon could be selected to perform the simulation. The T2 version [30] of Tersoff potential (Tersoff.mod) was selected due to its outstanding results on the melting and elastic characteristics of silicon [31]. This potential was developed by J. Tersoff in 1989, which aimed to reveal the elastic properties of silicon precisely. Accurate results [32] have been achieved describing silicon structures such as cluster, crystal orientation, liquid silicon and cubic diamond silicon in the molecular dynamics application of the Tersoff T2 potential. It also shows great adaptability in revealing crystal defects and internal stress. A comparison of the elastic constants obtained using different methods is plotted as Table 3. Among all the methods, obviously the molecular dynamics results achieved by the Tersoff T2 potential obtain the lowest disparity to the experimental values. The table demonstrates that the selection of the potential is accurate and appropriate for this simulation.

### 2.3. Method

After the simulation models were generated, these models were thermally equilibrated to 300 K for 200 picoseconds from their initial temperature via a NPT (isobaric/isothermal constant number of particles, constant pressure and constant temperature) ensemble. The pressure of the system was controlled close to 0 GPa in this process. The timestep was set to 1 femtosecond. Then, the simulation models started to load tensile strain, which was along the X axis of each model. To ensure the effects of strain rate were limited on the mechanical performance, the strain rate was set to 1 × 10^3^/ps^−1^. Except for the pressure of the X axis, the pressure of both the Y and Z axes were controlled close to 0 GPa via a Berendsen [38] barostat. The temperature of the system was controlled to 300K via a Berendsen thermostat in the whole deformation process. The trajectories of atoms were calculated using the Verlet algorithm. Ovito [39] software (Version 2.6.1) was used to create the original figures of the present paper. The atomic stress level (also called the virial stress) of each atom was calculated using the formula listed below:

(1) $$\sigma_{i}^{Atom}=\frac{1}{V^{Atom}}\left(-mv_{i}⊗v_{i}+\frac{1}{2}\sum_{j(\neq i)}r_{ij}⊗f_{ij}\right)$$

where the $\sigma_{i}^{Atom}$ is the level of virial stress, the $V^{Atom}$ is the volume of atom *i*, $v_{i}$ is the velocity of atom *i*, *r_ij_* is the relative displacement between atom *i* and *j*, *f_ij_* is the interatomic force and the symbol ⊗ represents the tensor product. According to this formula, the tensile stress level of each atom was calculated and visualized through Ovito. The calculation of atomic stress would help the discussions about the distribution and variation of internal stress. We used computer clusters to run the LAMMPS software and simulate the process. Three nodes with 120 CPU cores were used in each simulation case. For more details about the model and method, please refer to this article [28].

## 3. Results

### 3.1. Anisotropy of Mechanical Performances

The structural anisotropy of crystal will result in mechanical anisotropy in different crystal orientations. To measure the mechanical performance, parameters about the crystal mechanics were calculated and plotted as Table 4. We defined the “single defect decay” as a parameter which describes the resistance to the strength decay caused by a point defect with monoatomic size in each orientation. The value of the single defect decay is determined by the strength difference of the ideal simulation model and the simulation model with a monatomic defect. The orientation with the lowest value of the single defect decay obtains the highest strength resistance to the decay caused by the defect. The results of Table 4 show that the mechanical properties of [111] orientation are the highest and the mechanical properties of [100] orientation are the lowest, because the highest tensile strength is obtained for interfaces with the highest plane density and the lowest atomic disorder [40]. A higher yield strength profits the high-speed cutting as it increases the cutting efficiency. A lower single defect decay could gain potential advantages in the wafer cutting as it produces fewer defective products than other orientations with higher values.

**Figure 2:**
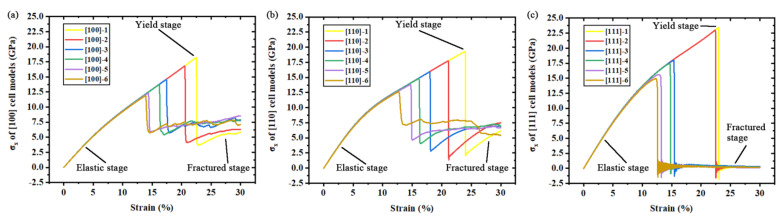
Stress-strain curves of all simulation models: (**a**) stress-strain curves of [100] simulation models; (**b**) stress-strain curves of [110] simulation models; (**c**) stress-strain curves of [111] simulation models.

The stress-strain curves of all simulation models are plotted as Figure 2a–c, which show the basic mechanical performances of three orientations in the uniaxial tensile deformation process. The curves are divided into three stages: elastic stage, yield stage and fractured stage. At the initial elastic stage in Figure 2, all simulation models exhibit the same crystallographic and mechanical performance, while no dislocations appear in these models. The evolutions of the curves (slopes of the curves) are determined by the anisotropic Young’s modulus in this stage. As we can see in the yield stage, three kinds of simulation models perform the anisotropic fracture phenomena and remain for a short period. The crystallographic anisotropy significantly affects yield strength and Young’s modulus when comparing curves of ideal crystal models from different orientations. Clearly, the existence of a defect cannot affect Young’s modulus of silicon. However, the increasing size of the point defect contributes to the reduction of yield strength. That means it is a kind of defect that decreases mechanical performance for wafer cutting while existing in the crystal alone. The rising trend of the lower yield point means that the level of residual stress is higher in silicon crystals with a larger point defect.

**Figure 3:**
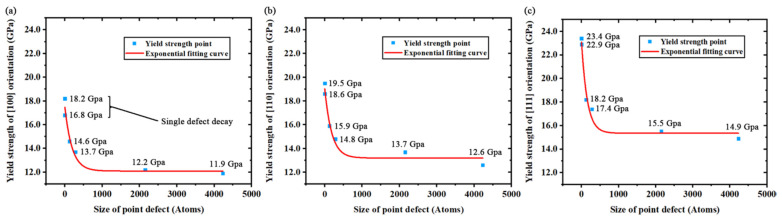
Yield strength variations of three kinds of simulation model caused by the size increase of point defect: (**a**) yield strength of [100] simulation models; (**b**) yield strength of [110] simulation models; (**c**) yield strength of [111] simulation models.

The variations of yield strength under the nanoscale effects of the point defect are shown in Figure 3 as the yield strength is a major concern in mechanical processing. The variations of strength in the [100], [110] and [111] orientations respectively match the exponential Formulas (2)–(4) listed below:

(2) $$\sigma_{\left[100\right]}(c)=\sigma_{1}+A_{1}\times Exp(R_{1}\times c),$$

(3) $$\sigma_{\left[110\right]}(c)=\sigma_{2}+A_{2}\times Exp(R_{2}\times c),$$

(4) $$\sigma_{\left[111\right]}(c)=\sigma_{3}+A_{3}\times Exp(R_{3}\times c),$$

where *σ*_[100]_, *σ*_[110]_ and *σ*_[111]_ represent the yield strength of the [100], [110] and [111] orientations respectively. *σ*_1_, *σ*_2_ and *σ*_3_ represent the minimum yield strength of each orientation respectively under the effects of a point defect. *c* is the atomic size of the point defect, measured by the amount of atoms. *A*_1_, *A*_2_, *A*_3_, *R*_1_, *R*_2_ and *R*_3_ are parameters related to the orientation and defect properties. *A* represents the difference between the minimum yield strength and the ideal yield strength. *R* represents the coefficient of the defect scale in the exponential fitting. The fitting formulas are selected with the clearest express, the highest correlation coefficient and the lowest error range. Further experiments and analysis are required to reveal the meanings of parameter *R* and the factors that affect parameter *A* and *σ*. Table 5 gives the fitting values of the parameters.

The results of the exponential formulas demonstrate that the nanoscale effects of the point defect caused an exponential reduction on the yield strength of monocrystalline silicon. In addition, the universality of the exponential reduction is proved in all major orientations of silicon with the anisotropic effects on the parameter values of the exponential fitting. However, another discovery of such exponential reduction is that there is a minimum yield strength (*σ*) in each orientation according to the formulas while increasing the size of the point defect to a large atomic scale. Obviously, the minimum yield strength is related to the macroscopic mechanical strength in some engineering applications. Although great disparity has already been proved between the numerical strength given by molecular dynamics simulation and actual yield strength [41], the exponential reduction still remains a reference value in various engineering applications like silicon anode [42].

### 3.2. Anisotropy of Fracture Phenomena

**Figure 4:**
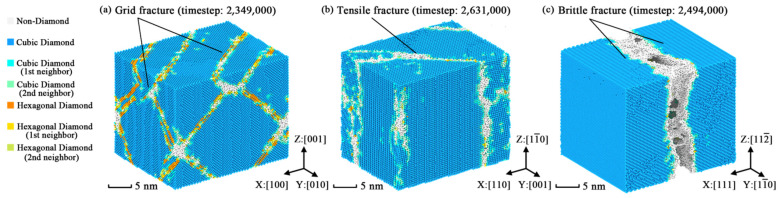
Fracture snapshots of three orientations under constant strain rate: (**a**) snapshot of [100] orientation; (**b**) snapshot of [110] orientation; (**c**) snapshot of [111] orientation.

The mechanical processing of silicon wafers led to a heterogeneous lateral strain distribution and various deformations of the silicon wafers. This would affect the resultant quality in the after-processing procedure [43]. Thus, the fracture phenomena should be clarified to understand the mechanical anisotropy. At the yield stage of Figure 2, cracks appear in the crystal models and extend to become crystal fractures at the lower yield point. The anisotropic fracture phenomena of three typical orientations are plotted as Figure 4 via Ovito.

**Figure 5:**
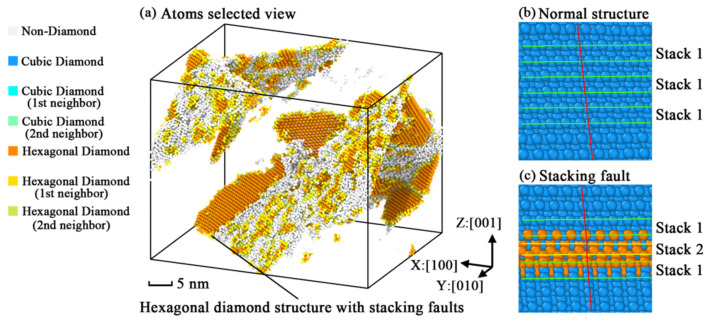
Geometric characteristics of fractured [100] simulation models: (**a**) atoms selected view of fracture plane without diamond silicon atoms; (**b**) structures with normal atomic arrangement in the (111) fracture plane; (**c**) structures with stacking faults in the (111) fracture plane.

**Figure 6:**
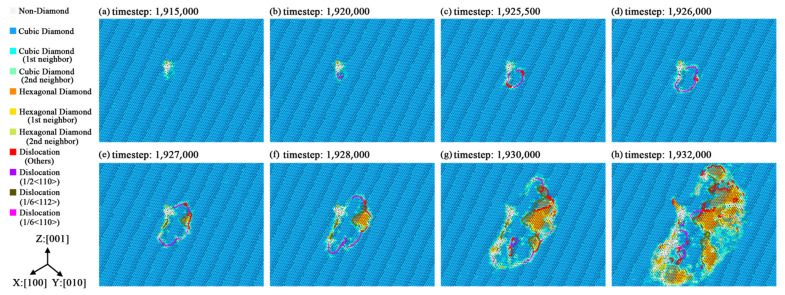
The dislocation movements at the (111) plane of simulation model [100]-4 in the fracture process: (**a**) point defect with structure distortion caused by the deformation at timestep 1,915,000; (**b**) dislocation nucleation nearby the point defect at timestep 1,920,000; (**c**) dislocation (1/2<110>) expanded at timestep 1,925,500; (**d**) dislocation (1/2<110>) expanded at timestep 1,926,000; (**e**) hexagonal diamond structures with stacking faults generated at timestep 1,927,000; (**f**) hexagonal diamond structures with stacking faults expanded at timestep 1,928,000; (**g**) dislocations slip along the (111) plane at timestep 1,930,000; (**h**) transection of cracks in the (111) plane at timestep 1,932,000.

Figure 4 indicates that all models perform three kinds of fractured structures in the fracture stage according to their orientations. That means the point defect cannot affect the anisotropic fracture phenomena. Figure 4a shows that two fracture planes (all belonging to the {111} plane family) with an angle of 70.53° appear in the crystal model of [100] orientation. In some degree, the fabrication of fractured planes is similar to the river pattern [44] found in the experiments of silicon fracture. It also looks like a grid structure [30] from the view of [110] orientation. Figure 4b points out that the yield fracture of the (111) plane is caused by strength-exceeded stress from the [110] orientation. For the [111] orientation, the fracture phenomenon is a kind of brittle fracture shown in Figure 4c. By observing the fractured structures shown in Figure 5, it is found that the fractured structures of [100] orientation are a hybrid of hexagonal diamond structures and non-diamond structures. The hybrid structures show typical stacking fault characteristics in Figure 5c compared with the normal silicon structures in Figure 5b. However, from the point of view of dislocation dynamics, the generation and expansion of stacking faults are considered as the slip of dislocation, which is along the slip planes of silicon. The discovery of stacking faults leads to the dislocation analysis about the simulation models presented in Figure 6.

Dislocation dynamics studies suggested that the crystallographic performance of silicon is related to the dislocation behaviors [45]. Research and direct observations [46,47] about silicon crystal have also indicated that the slip of dislocations is related to fracture and strength reduction. The DXA (dislocation analysis) function of the Ovito software was used to observe the dynamic evolutions of dislocations. The evolutions of dislocation in all simulation models are examined and some of them are plotted as Figure 6. Figure 6 shows the dislocation movements at the (111) plane of the simulation model [100]-4. The movements of dislocations in the fracture process are actually regarded as the slip, as they only appear in the cleavage planes of high plane density and create stacking faults. First, the dislocations are generated nearby the point defect in Figure 6b. The mechanism of this generation is similar to the conclusions from the research of Thaulow et al. [48]. Then, the dislocations spread along the <111> cleavage plane family to generate crystal cracks. As Figure 6e,f show, the hexagonal diamond structures and stacking faults are generated when the dislocations sweep across the (111) plane. The existence of hexagonal diamond structures at a certain visible scale is only found in the simulation models of [100] orientation. DXA also suggests that the formation and evolution of dislocations do not exist in the yield stages of [110] and [111] orientations. Therefore, we concluded that the dynamic slip of dislocation may be one of the reasons why the [100] orientation obtains the lowest yield strength among all major orientations. Zenari et al. [49] considered the point defect as the function of dislocation and their close relationships. Crystal defects (dislocation and point defect) are always related to each other closely in the view of crystallography. As for the nanoscale effects of the point defect, the formation of dislocation would be easier in those crystals with a point defect because of the structural distortions caused by the point defect. Such formation is harder for ideal crystals because they do not have any potential structural distortions, which provides an easier routine for the formation of dislocation actually.

**Figure 7:**
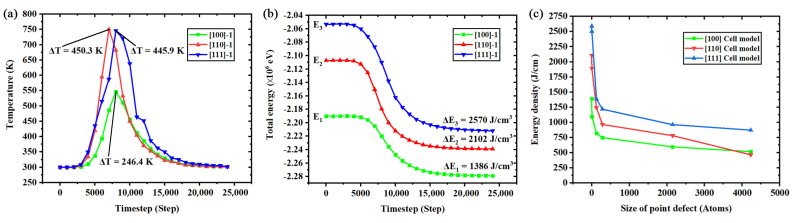
Temperature and total energy variation of the fracture process of the simulation models: (**a**) anisotropic temperature increase of three orientations; (**b**) anisotropic energy release of three orientations; (**c**) the variation of released energy density in the fracture process under the nanoscale effect of point defect.

Another interesting phenomenon found in the fracture stage is the release of elastic strain energy, which results in a sharp but anisotropic temperature increase shown in Figure 7a. For the temperature that was controlled by the Berendsen thermostat, the increase of temperature remained for a short duration. To reveal and discuss the mechanism of the anisotropic temperature increase, the variations of total energy and the energy densities released in the fracture process are plotted as Figure 7a,b. The released energies are converted to energy densities for unified comparison. The results suggest that the anisotropic temperature increase of the fracture process should be regarded as the anisotropic release of elastic strain energy. The anisotropy of elastic strain energy storage and release are the reasons for the maximum temperature differences in different orientations. Figure 7c shows the released energy density of each simulation model in the fracture process. As the figure shows, the curves of the three orientations exhibit obvious trends of exponential reduction due to the size increase of the point defect. Such a phenomenon was demonstrated in research about the phonon emission in the dynamic fracture process [50], because both of them could release the deformation energy stored in the microstructure which results in the increase of systematic temperature, and we guess that the increase of temperature includes the effects of phonon emission.

### 3.3. Anisotropy of Internal Stress

**Figure 8:**
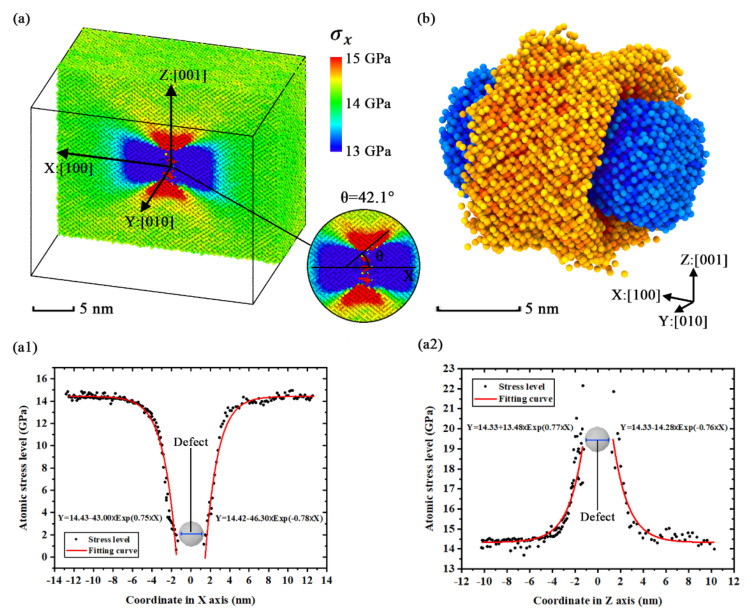
The distributions of *σ_x_* in simulation model [100]-4 with the average stress level of 5000 timesteps: (**a**) slice view along Z axis; (**b**) geometric characteristics of extreme stress region (*σ_x_* > 14.5 GPa or *σ_x_* < 13.5 GPa); (**a1**) numerical distribution of the atomic stress level in X axis (based on the coordinate shown in Figure 8a); (**a2**) numerical distribution of the atomic stress level in Z axis (based on the coordinate shown in Figure 8a).

**Figure 9:**
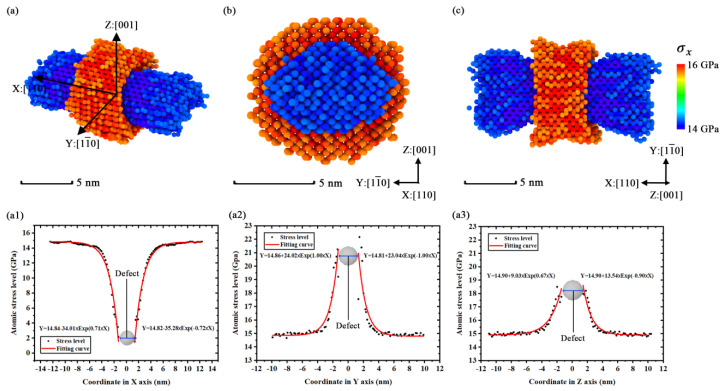
The distributions of *σ_x_* in simulation model [110]-4 with the average stress level of 5000 timesteps: (**a**) geometric characteristics of extreme stress region (*σ_x_* > 15.75 GPa or *σ_x_* < 14.25 GPa); (**b**) snapshot from [110] orientation; (**c**) snapshot from [001] orientation; (**a1**) numerical distribution of the atomic stress level in X axis (based on the coordinate shown in Figure 9a); (**a2**) numerical distribution of the atomic stress level in Y axis (based on the coordinate shown in Figure 9a); (**a3**) numerical distribution of the atomic stress level in Z axis (based on the coordinate shown in Figure 9a).

**Figure 10:**
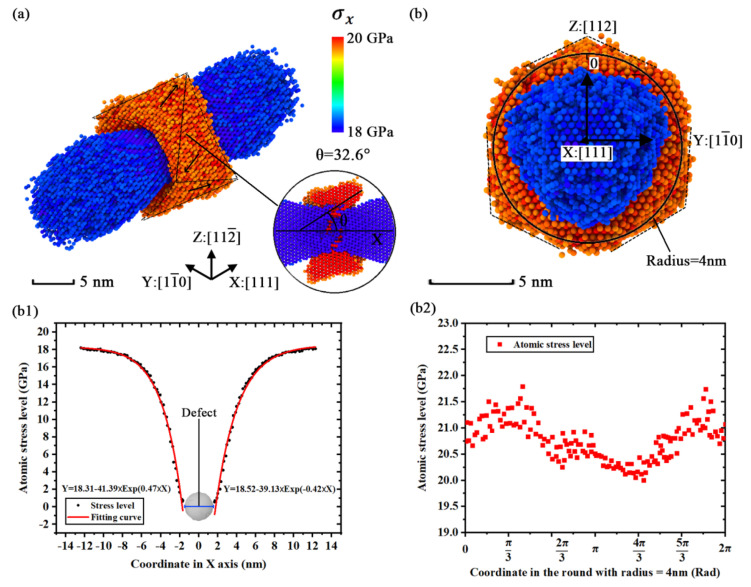
The distributions of *σ_x_* in simulation model [111]-4 with the average stress level of 5000 timesteps: (**a**) geometric characteristics of extreme stress region (*σ_x_* > 19.75 GPa or *σ_x_* < 18.25 GPa); (**b**) snapshot in the view of [111] orientation; (**b1**) numerical distribution of the atomic stress level in X axis (based on the coordinate shown in Figure 10b); (**b2**) numerical distribution of the atomic stress level in the round of YZ plane (based on the coordinate and round shown in Figure 10b, the radius of the round is 4 nm).

The internal stress performs different variations and distributions in different simulation models due to the crystallographic anisotropy. To verify the effects of internal stress on the fracture process, the stress distributions of some typical simulation models are plotted as Figure 8, Figure 9 and Figure 10 to analyze such results.

Figure 8a shows the distributions of stress concentration inside the simulation model [100]-4 via the slice modification of Ovito before the moment of fracture. Figure 8b shows the geometric characteristics of the extreme stress region by deleting the atoms with an average stress level. We find that the stress concentration is distributed as an annular region nearby the point defect, while the low-stress region is distributed like a dumbbell tied by the annular region of stress concentration. The stress concentration is focused on the defect in the simulation models ([100] orientation) with uniformity in the YZ plane due to the orientations in the Y axis and the Z axis being equivalent. In Figure 8(a1,a2), by calculating the atomic stress levels of the X and Y axes, it is clear that the atomic stress levels of the two axes exhibit trends of exponential reduction when the axial coordinates are close to the defect. The two figures show that the atomic stress level of the X axis is close to 0 GPa nearby the point defect and the atomic stress level of the Z axis is almost at 21 GPa nearby the point defect. With the coordinate value far away from the defect, the corresponding atomic stress level gradually closes to the average stress level. This indicates that the concentration factor of this model is nearly equal to 1.5.

The geometric characteristics of the extreme stress region in simulation model [110]-4 shown in Figure 9a are different from the simulation model [100]-4 because the orientations in its Y and Z axes are not equivalent. Figure 9b and c show that the shape of the extreme stress region looks like a squashed pillow. Except for the shape change of the extreme stress region, the variations of other properties such as general geometric shape and trend of atomic stress level variation are similar to the situation of simulation model [100]-4. However, by separately calculating the atomic stress level of the Y and Z axes in Figure 9(a2,a3), the distribution of the atomic stress level in simulation model [110]-4 exhibits obvious anisotropic performance in the YZ plane. For example, the concentration factor is 1.4 in the Y axis while the factor is 1.2 in the Z axis. A discrepancy of stress levels is found in different orientations.

The geometric characteristics of the extreme stress region in simulation model [111]-4 are plotted in Figure 10a. The characteristics show that the concentration region of internal stress is composed by several triangular structures. The crown-like structures exhibit characteristics of mirror symmetry and radiation symmetry in Figure 10a,b. The distribution of atomic stress in a round circle with a radius of 4 nm (almost the center of the annular region) was calculated and plotted in Figure 10(b2). The results of the atomic stress level in Figure 10(b2) show an obvious disparity in different azimuth, which gives us a basic understanding about the mechanical anisotropy. In the view of all visualized results of the internal stress, we concluded that the mechanical anisotropy affects the geometric characteristics of the extreme stress region in different simulation models. The geometric characteristics of the extreme stress region are related to the stress evolutions in the fracture process. Further visualizations suggest that the general characteristics of the extreme stress region are only determined by the direction of strain and the size of the point defect. The regional orientation is determined by the direction of strain and the entire regional scale relies on the defect size. That means a crystal cell could gain a more obvious concentration of extreme stress if there is a larger defect cluster inside.

**Figure 11:**
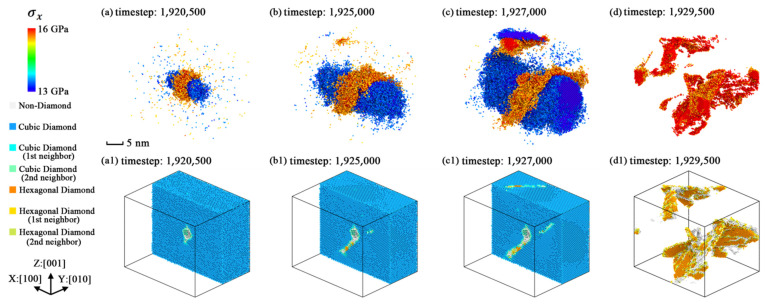
The geometric characteristics variation of extreme stress region in simulation model [100]-4 with the average stress level of 500 timesteps (*σ_x_* > 15.5 GPa or *σ_x_* < 13.5 GPa): (**a**) stress level selected snapshot at timestep 1,920,500; (**b**) stress level selected snapshot at timestep 1,925,000; (**c**) stress level selected snapshot at timestep 1,927,000; (**d**) stress concentration selected snapshot at timestep 1,929,500; (**a1**) slice view along Y axis at timestep 1,920,500; (**b1**) slice view along Y axis at timestep 1,925,000; (**c1**) slice view along Y axis at timestep 1,927,000; (**d1**) atoms selected view without diamond silicon atoms at timestep 1,929,500.

**Figure 12:**
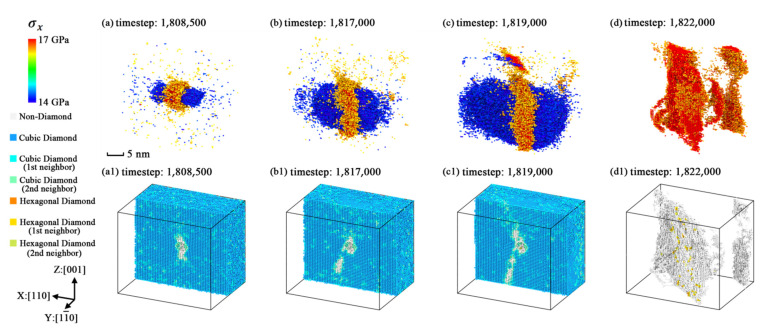
The geometric characteristics variation of extreme stress region in simulation model [110]-4 with the average stress level of 500 timesteps (*σ_x_* > 14.5 GPa or *σ_x_* < 16.5 GPa): (**a**) stress level selected snapshot at timestep 1,808,500; (**b**) stress level selected snapshot at timestep 1,817,000; (**c**) stress level selected snapshot at timestep 1,819,000; (**d**) stress concentration selected snapshot at timestep 1,822,000; (**a1**) slice view along Y axis at timestep 1,808,500; (**b1**) slice view along Y axis at timestep 1,817,000; (**c1**) slice view along Y axis at timestep 1,819,000; (**d1**) atoms selected view without diamond silicon atoms at timestep 1,822,000.

**Figure 13:**
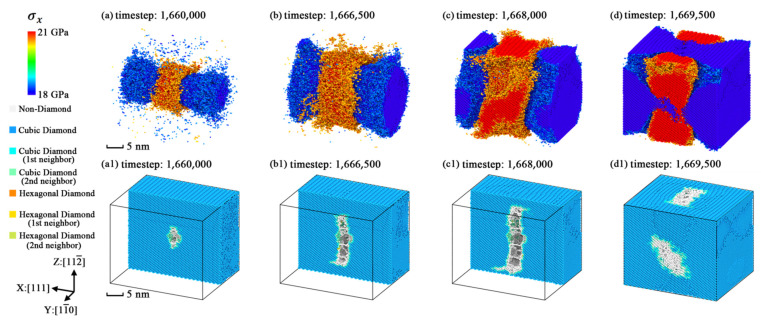
The geometric characteristics variation of extreme stress region in simulation model [111]-4 with the average stress level of 500 timesteps (*σ_x_* > 20.5 GPa or *σ_x_* < 18.5 GPa): (**a**) stress level selected snapshot at timestep 1,660,000; (**b**) stress level selected snapshot at timestep 1,666,500; (**c**) stress level selected snapshot at timestep 1,668,000; (**d**) stress concentration selected snapshot at timestep 1,669,500; (**a1**) Sslice view along Y axis at timestep 1,660,000; (**b1**) slice view along Y axis at timestep 1,666,500; (**c1**) slice view along Y axis at timestep 1,668,000; (**d1**) simulation model view at timestep 1,669,500.

The variations of the geometric characteristics of the extreme stress region are plotted as Figure 11, Figure 12 and Figure 13. The three figures indicate that the stress concentration is always distributed on the potential fracture path of each simulation model and the relationships between stress concentration and anisotropic fracture phenomena could be easily explained. From the dynamic evolution process in these figures, the structural fracture and stress concentration are considered to be mutually promoting each other. Thus, we regarded the stress concentration caused by the point defect as a reason for crystal fracture, because as the fractured structures appear in the crystal, the regions of stress concentration expand along the fractured structures of each model together with the regions of lower stress level expanding along the Y and Z axes.

## 4. Conclusions

The mechanical anisotropy of monocrystalline silicon under the nanoscale effects of a point defect was studied via general molecular dynamics methods. Based on the results, the following conclusions are made. 

The anisotropic mechanical performance suggests that the [100] orientation has the lowest mechanical performance while the [111] orientation has the highest yield strength and the maximum resistance to defect decay. The [111] orientation may gain potential advantages in diamond-saw cutting. By fitting the strength performance of all simulation models, we find that the variations of strength reduction caused by the point defect match the exponential relationships with defect size in all orientations. The parameters in the exponential fittings are affected by the mechanical anisotropy. The visualized fracture phenomena show that cleavage fracture, yield fracture and brittle fracture appear in the fracture stage of [100], [110] and [111] orientations respectively. Then, the DXA proves that only the fracture of [100] orientation is related to the slip movements of dislocations. The results of DXA indicate that the anisotropy of dislocations dynamics should be regarded as a reason for the fracture and the low strength performance of [100] simulation models. Another phenomenon of silicon anisotropy is the release of elastic strain energy in the fracture process. It leads to a temporal temperature increase. The calculation of energy indicates that the released energy density and defect size almost follow the exponential reduction, which proves the exponential universality of the nanoscale effects of a point defect on the fracture properties. Through the distributions of the internal stress, the extreme stress region exhibits special geometric characteristics in those models with point defects due to the mechanical anisotropy. One of the reasons for crystal fracture should be regarded as the variation of internal stress concentration, because the fracture and stress concentration are considered to be mutually promoting each other. The concentration factors of internal stress are affected by the mechanical anisotropy. Finally, the glaring issue here, though, is that the stress concentration is always distributed in the potential fracture path of each simulation model. This explains the relationships between stress concentration and anisotropic fracture phenomena.

The general anisotropic mechanical results under the nanoscale effects of a point defect could help us understand the fracture and mechanical anisotropy of monocrystalline silicon. It also provides a reference for the parameter optimization of diamond-wire cutting in photovoltaic applications.

## Figures and Tables

**Table 1:** Geometric parameters of three kinds of simulation model.

Model Type	Crystal Orientation			Axial Length/nm			Atoms Per Single Cell	Atoms Amount
	X Axis	Y Axis	Z Axis	X Axis	Y Axis	Z Axis		
[100] Simulation Model	[100]	[010]	[001]	21.7	21.7	21.7	8	512,000
[110] Simulation Model	[110]	[001]	$\left[1\bar{1}0\right]$	21.5	21.7	21.5	16	501,760
[111] Simulation Model	[111]	$\left[1\bar{1}0\right]$	$\left[11\bar{2}\right]$	21.6	21.5	21.3	32	494,592

**Table 2:** Postfix details about the simulation models.

Postfix of Model ID	“-1”	“-2”	“-3”	“-4”	“-5”	“-6”
Defect Size/Atoms	0	1	123	281	2149	4229
Defect Radius/nm	0.000	0.118	0.815	1.086	2.172	2.715

**Table 3:** The elastic constants of silicon obtained via different methods.

Elastic Constants	Expt. [33]	QM Methods		Molecular Dynamics Simulation			
		DFT [34]	TB [35]	Tersoff T2 [30]	Tersoff T3 [36]	SW [29]	EDIP [37]
*C*_11_ (GPa)	168	159	167	166	143	162	175
*C*_12_ (GPa)	65	61	67	65	75	82	62
*C*_44_ (GPa)	80	85	75	77	69	60	71

**Table 4:** Ideal mechanical performance of the major crystal orientations.

Crystal Orientation	Young’s Modulus/GPa	Yield Strength/GPa	Single Defect Decay/GPa
[100]	111.2	18.2	1.4
[110]	148.2	19.5	0.9
[111]	163.9	23.4	0.5

**Table 5:** Parameter values of Formulas (2)–(4).

Crystal Orientation	*σ*/GPa	*A*/GPa	*R*/Atoms^−1^
[100]	12.05 ± 0.46	5.41 ± 0.64	−0.0051 ± 0.0015
[110]	13.20 ± 0.44	5.82 ± 0.62	−0.0054 ± 0.0015
[111]	15.35 ± 0.47	7.74 ± 0.66	−0.0065 ± 0.0015

## Data Availability

Data available on request due to restrictions like data capacity. The data presented in this study are available on request from the corresponding author. The data are not publicly available due to data capacity (According to an uncompleted statistic, the related simulation data in the present paper is about at least 50 GB) which is too big to storage for the website. Please inform the corresponding author for further usage of the related simulation data.

## References

[1] Koga Y., Kurita K. (2021). Floating zone silicon wafer bonded to Czochralski silicon substrate by surface-activated bonding at room temperature for infrared complementary metal-oxide-semiconductor image sensors. Jpn. J. Appl. Phys..

[2] Kuo C.L., Nien Y.P., Chiang A.C., Atsushi H. (2021). Surface modification using assisting electrodes in wire electrical discharge machining for silicon wafer preparation. Materials.

[3] Kang R.K., Zeng Y.F., Gao S., Dong Z.G., Guo D.M. (2013). Surface layer damage of silicon wafers sliced by wire saw process. Adv. Mater. Res..

[4] Sekhar H., Fukuda T., Tanahashi K., Takato H., Ono H., Sampei Y., Kobayashi T. (2020). Mechanical strength problem of thin silicon wafers (120 and 140 µm) cut with thinner diamond wires (Si kerf 120→100 µm) for photovoltaic use. Mat. Sci. Semicond. Proc..

[5] Costa E.C., Xavier F.A., Knoblauch R., Binder C., Weingaertner W.L. (2020). Effect of cutting parameters on surface integrity of monocrystalline silicon sawn with an endless diamond wire saw. Sol. Energy.

[6] Borrero-Lopez O., Vodenitcharova T., Hoffman M. (2010). Anisotropy effects on the reliability of single-crystal silicon. Scripta Mater..

[7] Ebrahimi F., Kalwani L. (1999). Fracture anisotropy in silicon single crystal. Mat. Sci. Eng. A-Struct..

[8] Ebrahimi F., Hussain S.I. (1995). Crack path in single-crystals. Scr. Mater..

[9] Brookes C.A., Oneill J.B., Redfern B.A.W. (1971). Anisotropy in hardness of single crystals. Proc. R. Soc. Lond. Ser. A-Math. Phys. Sci..

[10] Perez R., Gumbsch P. (2000). Directional anisotropy in the cleavage fracture of silicon. Phys. Rev. Lett..

[11] Moulins A., Ma L.Y., Dugnani R., Zednik R.J. (2020). Dynamic crack modeling and analytical stress field analysis in single-crystal silicon using quantitative fractography. Theor. Appl. Fract. Mec..

[12] Mylvaganam K., Zhang L. (2014). Effect of crystal orientation on the formation of bct-5 silicon. Comp. Mater. Sci..

[13] George A., Michot G. (1993). Dislocation loops at crack tips: Nucleation and growth-an experimental study in silicon. Mat. Sci. Eng. A-Struct..

[14] Zhang X., Zhang L., Mironov S., Xiao R.S., Guo L., Huang T. (2021). Effect of crystallographic orientation on structural response of silicon to femtosecond laser irradiation. Appl. Phys. A-Mater..

[15] Liu B., Xu Z.W., Chen C., Li R., Gao X., Liang L. (2020). Numerical and experimental investigation on ductile deformation and subsurface defects of monocrystalline silicon during nano-scratching. Appl. Surf. Sci..

[16] Rickhey F., Marimuthu K.P., Lee K., Lee H. (2019). Indentation cracking of monocrystalline silicon considering fracture anisotropy. Theor. Appl. Fract. Mech..

[17] Liu X., Devor R.E., Kapoor S.G., Ehmann K.F. (2015). The mechanics of machining at the microscale: Assessment of the current state of the science. J. Manuf. Sci. Eng..

[18] Komanduri R., Raff L.M. (2001). A review on the molecular dynamics simulation of machining at the atomic scale. P. I. Mech. Eng. B-J. Eng..

[19] Goel S., Luo X.C., Agrawal A., Reuben R.L. (2015). Diamond machining of silicon: A review of advances in molecular dynamics simulation. Int. J. Mach. Tools Manuf..

[20] Gumbsch P., Zhou S.J., Holian B.L. (1997). Molecular dynamics investigation of dynamic crack stability. Phys. Rev. B.

[21] Wang G.L., Feng Z.J., Hu Y.H., Liu J., Zheng Q.C. (2020). Effects of anisotropy on single crystal silicon in polishing non-continuous surface. Micromachines.

[22] Wu H., Melkote S.N. (2013). Effect of crystal defects on mechanical properties relevant to cutting of multicrystalline solar silicon. Mat. Sci. Semicond. Proc..

[23] Korsós F., Roszol L., Jay F., Veirman J., Draoua D.A., Albaric M., Szarvas T., Kiss Z., Szabó A., Soczó I. (2018). Efficiency limiting crystal defects in monocrystalline silicon and their characterization in production. Sol. Energy Mater. Sol. Cell.

[24] Menold T., Hadjixenophontos E., Lawitzki R., Schmitz G., Ametowobla M. (2020). Crystal defects in monocrystalline silicon induced by spot laser melting. J. Appl. Phys..

[25] Pham V.T., Fang T.H. (2020). Anisotropic mechanical strength, negative Poisson’s ratio and fracture mechanism of borophene with defects. Thin Solid Films.

[26] Bagheripoor M., Klassen R. (2020). The effect of crystal anisotropy and pre-existing defects on the incipient plasticity of Fcc single crystals during nanoindentation. Mech. Mater..

[27] Dong S.H., Xia Y.X., Huang R.Y., Zhao J.H. (2019). Modulating mechanical anisotropy of two-dimensional materials by controlling their defects. Carbon.

[28] Wan W., Tang C.X., Qiu A., Xiang Y.K. (2021). The size effects of point defect on the mechanical properties of monocrystalline silicon: A molecular dynamics study. Materials.

[29] Balamane H., Halicioglu T., Tiller W.A. (1992). Comparative study of silicon empirical interatomic potentials. Phys. Rev. B.

[30] Tersoff J. (1988). Empirical interatomic potential for silicon with improved elastic properties. Phys. Rev. B.

[31] Zhou N.G., Hu Q.F., Xu W.X., Li K., Zhou L. (2013). A comparative study of different potentials for molecular dynamics simulations of melting process of silicon. Acta Phys. Sin-Ch. Ed..

[32] Zhou Z.Y., Wang T.B., Cheng Z.N. (1999). Molecular dynamics study on local structure of molten silicon. Acta Phys. Sin-Ch. Ed..

[33] Erhart P., Albe K. (2005). Analytical potential for atomistic simulations of silicon, carbon, and silicon carbide. Phys. Rev. B.

[34] Nielsen O.H., Martin R.M. (1985). Stresses in semiconductors: Ab initio calculations on Si, Ge, and GaAs. Phys. Rev. B.

[35] Lenosky T.J., Kress J.D., Kwon I., Voter A.F., Edwards B., Yang D.F., Yang S., Adams J.B. (1997). Highly optimized tight-binding model of silicon. Phys. Rev. B.

[36] Tersoff J. (1989). Modeling solid-state chemistry: Interatomic potentials for multicomponent systems. Phys. Rev. B.

[37] Justo J.F., Bazant M.Z., Kaxiras E., Bulatov V.V., Yip S. (1998). Interatomic potential for silicon defects and disordered phases. Phys. Rev. B.

[38] Berendsen H.J.C., Postma J.P.M., VanGunsteren W.F., DiNola A., Haak J.R. (1984). Molecular dynamics with coupling to an external bath. J. Chem. Phys..

[39] Stukowski A. (2010). Visualization and analysis of atomistic simulation data with ovito–the open visualization tool. Model. Simul. Mater. Sci. Eng..

[40] Amini H., Simchi A., Kokabi A.H. (2012). Effects of crystal orientation on the tensile and shear deformation of nickel-silicon interfaces: A molecular dynamics simulation. Mat. Sci. Eng. A-Struct..

[41] Tsai Y.L., Mecholsky J.J. (1991). Fractal fracture of single-crystal silicon. J. Mater. Res..

[42] Liu J., Yuan H. (2020). The evolution and failure mechanism of lithium metal anode under practical working conditions. J. Energy Chem..

[43] Herold S., Acker J. (2021). Lattice strain enhanced acidic etching on as cut sawn silicon wafer. Mat. Sci. Semicond. Proc..

[44] Chevrier J. (1995). Experimental-analysis of river patterns in silicon brittle fractures. J. Phys. I.

[45] Tsoutsouva M.G., Regula G., Ryningen B., Vullum P.E., Mangelinck-Noel N., Stokkan G. (2021). Dynamic observation of dislocation evolution and interaction with twin boundaries in silicon crystal growth using in-situ synchrotron X-ray diffraction imaging. Acta Mater..

[46] Sumino K., Yonenaga I. (1981). Difference in the mechanical strengths of dislocation-free crystals of Czochralski silicon and Float-zone silicon. Jpn. J. Appl. Phys..

[47] Chiao Y.H., Clarke D.R. (1989). Direct observation of dislocation emission from crack tips in silicon at high-temperatures. Acta Mater..

[48] Thaulow C., Sen D.P.J., Buehler M.J. (2011). Atomistic study of the effect of crack tip ledges on the nucleation of dislocations in silicon single crystals at elevated temperature. Mat. Sci. Eng. A-Struct..

[49] Zenari M., Buffolo M., De Santi C., Norman J., Meneghesso G., Bowers J.E., Zanoni E., Meneghini M. (2021). Identification of dislocation-related and point-defects in III-As layers for silicon photonics applications. J. Phys. D Appl. Phys..

[50] Atrash F., Hashibon A., Gumbsch P., Sherman D. (2011). Phonon emission induced dynamic fracture phenomena. Phys. Rev. Lett..

